# The Insufficient Number of Informative SNPs in a Preclinical Karyomapping Test for PGT-M Depends on the Reference Selected

**DOI:** 10.3390/jpm15070273

**Published:** 2025-06-26

**Authors:** Min Jee Kim, Yeseul Hong, Gaeul Han, Hyoung-Song Lee, Eun A. Park, Kyung-Ah Lee, Eun Jeong Yu, Inn Soo Kang

**Affiliations:** 1 Department of Biomedical Sciences, College of Life Sciences, CHA University, Seongnam 13488, Republic of Korea; mjkim44@chamc.co.kr (M.J.K.); leeka@cha.ac.kr (K.-A.L.); 2 Laboratory of Reproductive Genetics, CHA Biotech, Seoul Station, Seoul 04637, Republic of Korea; hyeslh1209@chamc.co.kr (Y.H.); autumnmon@chamc.co.kr (G.H.); hslee99@chamc.co.kr (H.-S.L.); 3 Fertility Research Lab, CHA Fertility Center Seoul Station, Seoul 04637, Republic of Korea; 4 Department of Obstetrics and Gynecology, CHA University School of Medicine, CHA Fertility Center Seoul Station, Seoul 04637, Republic of Korea; 5 Department of Obstetrics and Gynecology, CHA University School of Medicine, CHA Fertility Center Daegu, Daegu 41936, Republic of Korea; ikang@cha.ac.kr

**Keywords:** preimplantation genetic testing for monogenic disorders, karyomapping, single-nucleotide polymorphism, genome-wide single-nucleotide

## Abstract

**Background/Objectives**: Karyomapping, a genome-wide SNP analysis, has drastically changed the approach to preimplantation genetic testing for monogenic disorders (PGT-M). However, there are cases in which karyomapping cannot be applied due to an insufficient number of informative SNPs. In this study, we aimed to analyze for the first time whether an insufficient number of informative SNPs is related to the family member used as a reference. **Methods**: For the karyomapping pre-clinical test, in addition to the couple, one of the DNA samples from an additional family member (children, parent, sibling) is used as a reference for phasing the SNP allele. We analyzed 263 couples who underwent karyomapping for PGT-M at the CHA Fertility Center from May 2020 to December 2022. karyomapping data was scanned on an Illumina NextSeq and analyzed through the BlueFuse Multi software version 4.5. **Results**: Preclinical karyomapping tests were performed in 263 couples with 58 monogenic diseases. Karyomapping was applicable to PGT-M for 241 (91.6%) couples and not applicable for 22 (8.4%) couples. The percentages of “not applicable” cases according to the reference family member were 1.3% (1/80) in the children group, 5.4% (8/148) in the parent group, and 37.1% (13/35) in the sibling group. Among the genetic diseases studied, couples with neurofibromatosis type 1 (6/27, 22.2%) and Kennedy disease (5/5, 100%) had the highest rate of non-applicable cases. **Conclusions**: Our results suggest that a child or parent may be better than the sibling for karyomapping in PGT-M. These data provide useful information for selecting a reference among the family members for preclinical karyomapping tests.

## 1. Introduction

Preimplantation genetic testing for monogenic disorders (PGT-M) has been successfully used as an alternative to prenatal testing to prevent couples with a family history of specific genetic disorders from passing these disorders on to their child. The use of PGT-M allows high-risk patients to avoid the termination of an unaffected pregnancy or the birth of an affected child [1,2,3].

Diagnostic methods for identifying pathogenic or likely pathogenic variants in PGT-M include conventional polymerase chain reaction (PCR), multiplex PCR, single-nucleotide polymorphism (SNP) array, or next-generation sequencing (NGS). The main diagnostic method is targeted amplification with multiplex PCR to identify pathogenic or likely pathogenic variants and genetic markers. Other diagnostic methods are based on the principle of haplotyping, in which the risk-related haplotypes associated with a specific pathogenic or likely pathogenic variant are determined by observing how selected informative markers (SNPs or short tandem repeats (STRs)) segregate from the pathogenic or likely pathogenic variants after whole-genome amplification [4].

The conventional PGT-M method generally involves targeted amplification from a minuscule amount of DNA using PCR, sometimes resulting in allelic dropout (ADO), in which one of the parental alleles is randomly not amplified; ADO occurs in a low percentage of single cells but can lead to diagnostic errors. PGT-M is also susceptible to contamination due to the extreme sensitivity of its amplification and detection methods [5,6,7]. To avoid these potential sources of diagnostic errors, the current standard method is multiplex PCR for one or more polymorphic markers linked to the pathogenic or likely pathogenic variants of interest. STR markers close to the gene of interest enable more accurate diagnosis through the identification of parental haplotypes based on the chromosome affected by the pathogenic or likely pathogenic variants [7,8,9]. However, conventional PCR-based PGT-M using STR markers provides limited information near the target locus, and heterozygous STR markers must be assessed via capillary electrophoresis using premade kits. Additionally, the mutant and normal haplotypes used in multiplex PCR are established in the preclinical setting, and optimizing multiplex PCR at the single-cell level requires a significant amount of laboratory work and takes approximately 6 months [10,11].

Recently, karyomapping has been introduced as an alternative to conventional PGT-M. Karyomapping is based on linkage analysis using SNPs. SNPs are more frequent in the genome compared to STRs and are, therefore, more informative in this context. Therefore, whenever possible, SNP analysis should be preferred over STR analysis for indirect analysis [12,13,14]. Karyomapping is performed by genotyping parents and embryos for more than 300,000 SNPs across the entire genome. This method enables the application of a “genome-wide association analysis” approach to the diagnosis of single-gene disorders without the need for designing patient-specific assays. As a result, it reduces the time and labor associated with single-cell multiplex PCR optimization, which typically requires extensive effort and may take up to six months [15,16]. In addition, ADO, which is one of the most challenging problems of the conventional PCR method, can be overcome with a universal test using genome-wide SNP markers and haplotyping at the pathogenic or likely pathogenic variants site and adjacent regions [17]. Therefore, karyomapping for monogenic disorders is a faster, more efficient, and more reliable method than conventional PGT-M [18].

However, the karyomapping strategy for monogenic disorders faces several limitations. First, the preclinical testing stage of karyomapping requires appropriate reference samples. Informative SNPs associated with the mutant allele in the embryo are identified using a DNA sample obtained from an additional family member, at least a first-degree relative (children, parent, sibling) with the same pathogenic or likely pathogenic variants. For some couples, it is difficult to perform preclinical karyomapping testing due to a lack of affected family members; in these cases, DNA from an unaffected family member can be used as a reference. By using this unaffected family member, it is possible to infer the normal haplotype of the affected partner, thereby enabling the identification of the normal genotype in the embryo. Additionally, one or more DNA samples obtained from affected embryos or sperm from a previous in vitro fertilization (IVF)/PGT for structural rearrangements (PGT-SR) cycle could be used as a reference if the quality were sufficient [16,19]. However, an appropriate reference may not be available; without reference DNA, it is difficult to perform karyomapping due to SNP informativeness being reduced [20]. Second, karyomapping cannot be applied to patients with de novo pathogenic or likely pathogenic variants because no family member can be used as a reference. Third, genome-wide recombination is inevitable, and accurate SNP linkage analysis may not be possible if chromosomal recombination occurs in the causative gene.

The location and number of informative SNPs are very important for karyomapping diagnosis and improve the accuracy of karyomapping. However, the acceptability of karyomapping depends on the distance of the informative SNPs from the region of interest and whether family samples are available to obtain sufficient SNP data for phasing [20].

Although there are a sufficient number of SNPs associated with most genes of interest, there are cases in which karyomapping is not applicable due to an insufficient number of informative SNPs. In these cases, conventional PCR-based PGT-M methods (e.g., direct DNA sequencing and/or linkage analysis) should be performed in parallel with karyomapping to confirm the results. We wondered whether an insufficient number of informative SNPs was related to the family member used as a reference. To date, there has been no report comparing the number of informative SNPs according to the reference among family members and confirming their application rate. In this study, we aimed to investigate the relationship between the lack of informative SNPs and the family member used as a reference.

## 2. Materials and Methods

### 2.1. Subjects

This retrospective study included 263 couples who underwent genome-wide SNP analysis via Karyomapping for PGT-M at CHA Biotech, Seoul Station, from May 2020 to December 2022. Fifty-eight different monogenic disorders, including autosomal dominant, autosomal recessive, and X-linked disorders, were evaluated (Supplementary Table S1). For the karyomapping test, the DNA of the couple and one first-degree relative (child, parent, or sibling) who has the same genetic condition as the affected partner was used as a reference for phasing SNP alleles. In cases where parental divorce, loss of contact, or refusal to provide DNA samples occurred, alternative reference individuals were selected. The references were divided into three groups: the children group, the parent group, and the sibling group. Appropriate genetic counseling was provided to all couples who were considering PGT-M. Informed consent was obtained from all participants.

### 2.2. Preclinical Test for Karyomapping

Before the preclinical test, DNA was extracted from the EDTA-treated peripheral blood of all family members within one week after blood collection using a Quickgene DNA Whole Blood Kit according to the manufacturer’s instructions (SHIMADZU, Neyagawa, Japan). The extracted DNA was used for the preparation of karyomapping and for the direct sequencing or linked STR markers for patients with variants. For the preclinical test, DNA samples of reference were obtained from the couple’s son/daughter (children group) or parents (parent group), brother/sister (sibling group) karyomapping was performed using an Infinium Human Karyomap-12 DNA analysis kit (Cat#1500055; Illumina, San Diego, CA, USA), and fragmentation, precipitation, resuspension, hybridization, washing, and staining were performed according to the manufacturer’s instructions.

### 2.3. Karyomapping Analysis

Karyomapping data were scanned on an Illumina NextSeq (Illumina, San Diego, CA, USA) and analyzed through the BlueFuse Multi software version 4.5 (Illumina). SNP genotype data from children, parents, and sibling relatives were used for karyomapping analysis. For genomic DNA samples, call rates of more than 98% were typically achieved. The combination of parental genotypes at each SNP locus was analyzed to identify informative loci where one parent was homozygous (AA or BB) and the other parent was heterozygous (AB). The genotypes of affected family members (children, parents, sibling) with the same pathogenic or likely pathogenic variants were used as a reference, the mutant allele on one of the four parental chromosomes was identified, and a haploblock was generated using the informative SNPs of the male partner and female partner.

Haplotypes generated via karyomapping were associated with the reference sample. Blue and orange represent paternal and maternal haplotypes, respectively, inherited via the reference, and red and green represent non-inherited paternal and maternal haplotypes, respectively. Phasing with the reference sample allowed the identification of the mutant allele in the haploblock; in addition, heterozygous SNP reads in the 2 Mb region in the 5′ and 3′ flanking regions of the targeted gene were used to identify mutant alleles [15].

To apply Karyomapping for PGT-M, there should be ≥5 informative SNPs in the 5′ flanking region and 3′ flanking region. If there are fewer than 5 informative SNPs, the case is still considered “applicable” if the SNPs are evenly distributed in one or both regions and informative SNPs exist in the main regions. If there are no informative SNPs in one or both flanking regions and no intragenic informative SNPs in the main region, karyomapping was considered “not applicable” for PGT-M, and PCR-based direct sequencing, and linkage analysis were performed.

## 3. Results

A preclinical karyomapping test was performed for 263 couples with 58 monogenic disorders. The average SNP call rate of DNA samples was 98% (Illumina’s recommended SNP call rate, >95%), which is considered sufficient for karyomapping analysis. Among the 263 couples, karyomapping was applicable for 241 couples (91.6%) and was not applicable for 22 couples (8.4%).

For the monogenic disorders included in our study, the mean number of available SNPs was 450 (including the gene of interest and 2 Mb on either side of the gene), and the mean number of informative SNPs was 52.9 (Supplementary Table S1). Five gene regions were found to have relatively low SNP coverage (less than 130 available SNPs): *COL7A1* (72 SNPs, epidermolysis bullosa, OMIM:131750), *POU3F4* (101 SNPs, hereditary deafness, OMIM:304400), *AR* (114 SNPs, Kennedy disease, OMIM: *313700), *ATP7A* (118 SNPs, Menkes syndrome, OMIM: 309400), and *SMN1* (120 SNPs, spinal muscular atrophy, OMIM: 253300).

**Figure 1:**
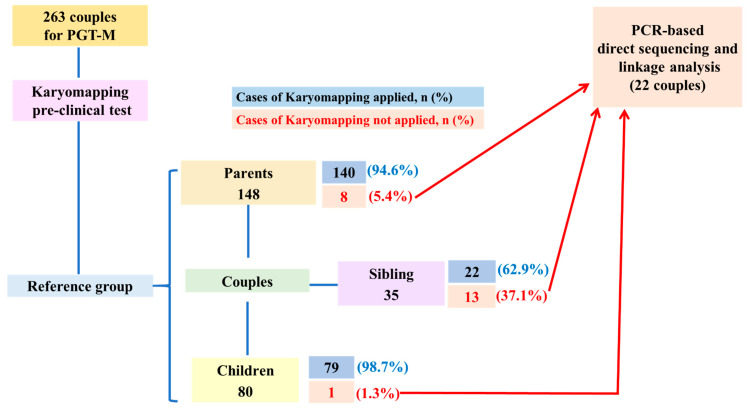
Comparison of Karyomapping-applicable cases according to the reference chosen. The number of successful Karyomapping cases is indicated in the blue boxes, with the corresponding percentages shown in parentheses. The number of unsuccessful Karyomapping cases is indicated in the skin-colored boxes, also with percentages shown in parentheses. PCR, polymerase chain reaction; PGT-M, preimplantation genetic testing for monogenic disorders.

As shown in Figure 1, the proportion of cases for which karyomapping was not applicable was greater in the sibling group (37.1%, 13/35) than in the children group (1.3%, 1/80) and the parent group (5.4%, 8/148). The mean number of informative SNPs (Table 1) was greater in the children group (87 informative SNPs) than in the parent group (42 informative SNPs) and sibling group (39 informative SNPs). In the sibling group only, there were 10 patients with fewer than 5 SNPs for which information was insufficient for PGT-M. Karyomapping was not applicable for 11 monogenic disorders (Table 2). The most common disorder was Kennedy disease in five couples (5/5, 100.0%), followed by neurofibromatosis type 1 (NF1) in six couples (6/27, 22.2%). The disease with the lowest numbers of informative SNPs and available SNPs was Kennedy disease, with 114 available SNPs and an average of 28.8 informative SNPs.

Table 3 shows the cases with an insufficient number of informative SNPs stratified according to the karyomapping reference for PGT-M. In 9 of 22 couples (41%), no informative SNPs were found in the 5′ region, main region, or 3′ region (two cases each with Charcot–Marie–Tooth disease (CMT) and myotonic dystrophy 1 (DM1) and one case each with von Hippel–Lindau syndrome, Duchenne muscular dystrophy, Marfan syndrome, spinocerebellar ataxia, and ornithine transcarbamylase deficiency. The reference family members used in these nine cases were all siblings (from the same generation as the couple). Siblings were selected as alternative references due to circumstances such as parental divorce, loss of contact, or parental refusal to provide a DNA sample (Supplementary Figure S1). In the other 13 patients, informative SNPs were found only in the 5’ flanking region (1 patient each with hemophilia A and Fabry disease) or the 3’ flanking region (6 patients with NF1 and 5 patients with Kennedy disease).

In particular, androgen receptor (AR) genes, the causative genes for Kennedy disease (Supplementary Table S2), had a much lower number of available SNPs (114 SNPs), with an average of 28.8 informative SNPs per couple (five couples total). Moreover, the 5′ flanking regions were not covered in all five couples (none of the 25 available SNPs were informative SNPs). In these cases, informative SNPs were not evenly distributed, and there were no intragenic informative SNPs within the main regions. Therefore, the 22 couples who were considered “not applicable” for Karyomapping for PGT-M in the preclinical test were diagnosed using PCR-based direct sequencing and linkage analysis using STR markers for PGT-M.

## 4. Discussion

To our best knowledge, this is the first study to identify an insufficient number of informative SNPs according to the family member used as a reference for Karyomapping in PGT-M.

In this study, we evaluated cases with an insufficient number of informative SNPs according to the family member used as a reference and cases of genetic diseases in which karyomapping for PGT-M was not applicable.

Our results showed that the mean number of informative SNPs in the sibling reference group (39 informative SNPs) was lower than that in the children group (87 informative SNPs) and parent group (42 informative SNPs). There were more cases in which karyomapping could not be applied in the sibling reference group (37%) than in the children or parent group. There are more patients with fewer than five informative SNPs that were insufficient for karyomapping in the sibling group (10 patients) compared to none in the children or parent group. This suggests that a child or parent may be the better reference for karyomapping for PGT-M than the sibling in karyomapping for PGT-M.

**Figure 2:**
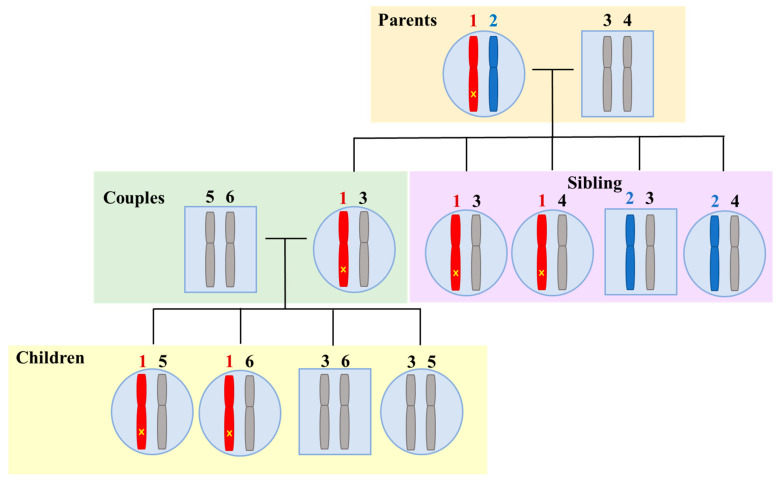
Allele sharing at genetic locus between siblings for a disease. Alleles are represented with numbers (1–6). The yellow ‘x’ on the red chromosome inherited from the female partner’s mother represents the mutant allele, while the blue chromosome represents the normal allele. Siblings (including dizygotic twins) share 50% of their alleles with their other siblings; however, a quarter of siblings (25%) inherit the same two alleles at a particular gene locus.

This finding can be explained by applying the principle of allele sharing among relatives: a quarter of siblings inherit two identical alleles at a particular locus, a quarter have no common allele, and half share one allele [21]. Therefore, a quarter of the siblings, who are the sisters or brothers of the affected couple, used as a reference are likely to share the same allele according to the segregation principle (Figure 2). As a result, the number of informative SNPs for the parental alleles carrying the pathogenic or likely pathogenic variants was close to zero, and there were many non-informative SNPs. It has been reported that it can be difficult to apply association analysis to noninformative SNPs due to the high risk of erroneous results such as ADO [17].

Using the parents or other relatives of one person in a couple as a reference allows the phasing of SNP loci only for that partner. For non-children reference, only loci for which the reference was homozygous are shown. This eliminates ambiguity in the haplotypes of references that were not inherited by the couple and reduces the amount of available data by an average of ~50% [16]. On the other hand, the children group, in which the couple’s offspring were used as references, showed a higher number of informative SNPs compared to other groups. A stepwise analysis of SNP information from both the female and male partners in PGT-M allowed for the more precise identification of embryo genotypes. In cases where the number of these informative SNPs was insufficient, we determined whether sufficient informative SNP data could be obtained using additional family member samples; if additional family samples were not available, PCR-based direct sequencing and linkage analysis were performed.

As previously reported, Karyomapping chips were evaluated to determine whether they provided adequate SNP coverage for genomic regions [16,22,23]. However, Konstantinidis [22] reported that some regions were less covered; cystic fibrosis (7q31.2; mean available SNPs: 182; mean informative SNPs: 29.5) and spinal muscular atrophy (5q13.2; mean available SNPs: 134; mean informative SNPs: 10.5) had lower SNP coverage compared to other diseases (mean available SNPs: 515.9; mean informative SNPs: 73.8). Additionally, it has been reported that these genetic disorders are difficult to diagnose via karyomapping [22]. On the other hand, in our study, Kennedy disease was found to be more challenging to diagnose with karyomapping than the other diseases. In five couples with Kennedy disease, there were no informative SNPs in the 5’ flanking region of the AR gene, which is thought to be due to the small number of SNPs in the centromeric region of the X chromosome. However, there were sufficient available and informative SNPs for causative genes of other X-linked diseases (e.g., hemophilia A and fragile X syndrome), regardless of location (Supplementary Table S2). Another method of addressing this issue for non-telomeric genes is to expand the upstream and downstream evaluation regions of the gene from 2 Mb to 3 Mb. This should increase the number of available SNPs and will likely also increase the number of informative SNPs. In addition, if the SNP coverage of a gene is relatively low but sufficient intragenic SNPs exist in the main region, these intragenic SNPs, which have great value in accurate diagnosis, can potentially compensate for the lack of SNPs in the flanking regions. On the other hand, in the case of the AR gene, extending the 5’ flanking region to 3 Mb did not increase the number of available SNPs, and no intragenic SNPs were found within the gene. Therefore, direct sequencing and STR analysis were performed together to compensate for the insufficient number of SNPs in the 5’ flanking region. This study involved several limitations that should be acknowledged. It was conducted exclusively on patients in Korea who underwent preimplantation genetic testing for single-gene disorders, which may limit the generalizability of the results to broader populations. To enhance the validity and applicability of these findings, future studies involving larger sample sizes and more diverse, multicenter cohorts will be necessary.

## 5. Conclusions

Karyomapping is an advanced method compared to the existing PCR methods in PGT-M. In the preclinical stage of karyomapping, when the reference family member was a sibling, the probability that karyomapping could not be used was greater than when the reference family member was a parent or child. To increase the usefulness of karyomapping, it is important to carefully review family history and select an appropriate reference through genetic counseling. Our study is the first to identify an insufficient number of informative SNPs according to the family member used as a reference in PGT-M via karyomapping. These data would provide useful information for selecting references for karyomapping preclinical tests.

## Figures and Tables

**Table 1:** Mean number of informative SNPs according to the reference chosen.

Reference	Children	Parent	Sibling
Number of cases	80	148	35
KM applicable, *n* (%)	79 (99)	140 (95)	22 (63)
Not applicable, *n* (%)	1 (1)	8 (5.4)	13 (37)
Mean number of informative SNPs			
5′ region	39	22	21
Main region	4	3	6
3′ region	44	17	12
Total	87	42	39
Number of cases < 5 informative SNPs	0	0	10

SNP, single-nucleotide polymorphism; KM, karyomapping.

**Table 2:** Number of informative SNPs for monogenic disorders not applicable to karyomapping.

Disease	Not Applicable (%, Per Couple)	Inheritance Mode	Gene	Locus	No. of Available SNPs	Mean No. of Informative SNPs
Neurofibromatosis, type I (NF1)	6/27 (22.2)	AD	*NF1*	17q11.2	616	45.9
Kennedy disease	5/5 (100)	X-linked	*AR*	Xq12	114	28.8
Charcot–Marie–Tooth disease, type 1A (CMT1A)	2/32 (6.3)	AD	*PMP22*	17p12	599	37.1
Myotonic dystrophy 1 (DM1)	2/24 (8.3)	AD	*DMPK*	19q13.32	243	31.1
von Hippel–Lindau syndrome (VHL)	1/6 (17)	AD	*VHL*	3p25.3	406	37.1
Marfan syndrome (MFS)	1/12 (8.3)	AD	*FBN1*	15q21.1	549	44.8
Spinocerebellar ataxia (SCA)	1/4 (25)	AD	*ATXN3*	14q32.12	402	36
Hemophilia A (HEMA)	1/14 (7.1)	X-linked	*F8*	Xq28	550	45.8
Fabry disease	1/2 (50)	X-linked	*GLA*	Xq22.1	593	44
Duchenne muscular dystrophy (DMD)	1/12 (8.3)	X-linked	*DMD*	Xp21.2-p21.1	656	100
Ornithine transcarbamylase deficiency (OTC)	1/3 (33.3)	X-linked	*OTC*	Xp11.4	421	60.4

AD, autosomal dominant; *PMP22*, peripheral myelin protein 22; *AR*, androgen receptor; *DMPK*, dystrophia myotonica protein kinase; *VHL*, von Hippel–Lindau; *FBN1*, fibrillin 1; *ATXN3*, ataxin 3; *F8*, coagulation factor VIII; *GLA*, galactosidase alpha; SNP, single-nucleotide polymorphism.

**Table 3:** Cases with an inadequate number of informative SNPs according to the Karyomapping reference for PGT-M.

Case No.	Disease	Inheritance Mode	Gene	Affected Partner of the Couple	Family Member Selected as a Reference	Genetic Status of Reference	Informative SNPs		
							5′ Region	Main Region	3′ Region
1	VHL	AD	*VHL*	Male	Sibling (brother)	Affected	0	0	0
2	CMT1A	AD	*PMP22*	Female	Sibling (sister)	Affected	0	0	0
3	CMT1A	AD	*PMP22*	Female	Sibling (sister)	Affected	0	0	0
4	DM1	AD	*DMPK*	Male	Sibling (brother)	Affected	0	0	0
5	DM1	AD	*DMPK*	Male	Sibling (sister)	Affected	0	0	0
6	DMD	X-linked	*DMD*	Female	Sibling (sister)	Carrier	0	0	0
7	MFS	AD	*FBN1*	Male	Sibling (brother)	Affected	0	0	0
8	SCA	AD	*ATXN3*	Male	Sibling (sister)	Affected	0	0	0
9	OTC	X-linked	*OTC*	Female	Sibling (sister)	Affected	0	0	0
10	NF1	AD	*NF1*	Male	Sibling (sister)	Affected	0	0	3
11	NF1	AD	*NF1*	Female	Sibling (sister)	Affected	0	0	7
12	NF1	AD	*NF1*	Female	Sibling (brother)	Affected	0	0	7
13	HEMA	X-linked	*F8*	Female	Sibling (brother)	Affected	36	0	0
14	NF1	AD	*NF1*	Male	Children	Affected	0	0	13
15	NF1	AD	*NF1*	Male	Grandfather	Affected	0	0	7
16	NF1	AD	*NF1*	Male	Grandmother	Affected	0	0	16
17	Fabry disease	X-linked	*GLA*	Female	Grandmother	Carrier	32	0	0
18	Kennedy disease	X-linked	*AR*	Female	Grandfather	Affected	0	0	23
19	Kennedy disease	X-linked	*AR*	Female	Grandfather	Affected	0	0	27
20	Kennedy disease	X-linked	*AR*	Female	Grandfather	Affected	0	0	33
21	Kennedy disease	X-linked	*AR*	Female	Grandfather	Affected	0	0	33
22	Kennedy disease	X-linked	*AR*	Female	Grandfather	Affected	0	0	28

VHL, von Hippel–Lindau; CMT1A, Charcot–Marie–Tooth disease type 1A; DM1, myotonic dystrophy 1; DMD, Duchenne muscular dystrophy; MFS, Marfan syndrome; SCA, spinocerebellar ataxia; OTC, ornithine transcarbamylase deficiency; NF1, neurofibromatosis, type 1; HEMA, hemophilia A; AD, autosomal dominant; *PMP22,* peripheral myelin protein 22; *DMPK*, dystrophia myotonica protein kinase; *FBN1,* fibrillin 1; *ATXN3,* ataxin 3; *F8*, coagulation factor VIII; *GLA,* galactosidase alpha; *AR*, androgen receptor; SNP, single-nucleotide polymorphism; PGT-M, preimplantation genetic testing for monogenic disease.

## Data Availability

The datasets generated and analyzed during the current study are not publicly available due to a concern for protecting individual patient confidentiality, but they are available from the corresponding authors upon reasonable request.

## Supplementary Materials

The following supporting information can be downloaded at https://www.mdpi.com/article/10.3390/jpm15070273/s1, Table S1. This table shows the number of available SNPs and the mean number of informative SNPs according to genetic disease (Microsoft Excel 2019). Table S2. This table shows the mean number of available SNPs and informative SNPs in X-linked disease (Microsoft Excel 2019). Figure S1. Pedigrees of cases with an inadequate number of informative SNPs according to the karyomapping reference for PGT-M (Microsoft Word 2019).

## Abbreviations

The following abbreviations are used in this manuscript:

PGT-M	Preimplantation genetic testing for monogenic disorders
PCR	Polymerase chain reaction
SNP	Single-nucleotide polymorphism
STR	Short tandem repeat
ADO	Allelic dropout
IVF	In vitro fertilization
PGT-SR	Preimplantation genetic testing for structural rearrangements
CMT	Charcot–Marie–Tooth disease
DM1	Myotonic dystrophy 1
