# Supplementary material for: The Insufficient Number of Informative SNPs in a Preclinical Karyomapping Test for PGT-M Depends on the Reference Selected
Source: J Pers Med. 2025 Jun 26;15(7):273. doi: 10.3390/jpm15070273 (PMC12300894; doi:10.3390/jpm15070273)

**1. VHL (VHL gene)**

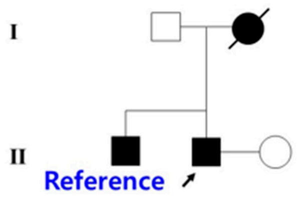


**2. CMT1A (PMP22 gene)**

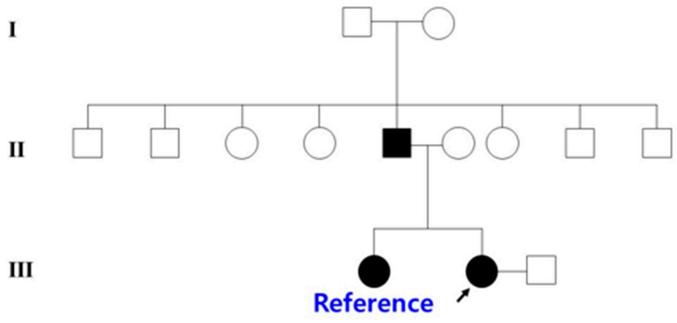


**3. CMT1A (GJB1 gene)**

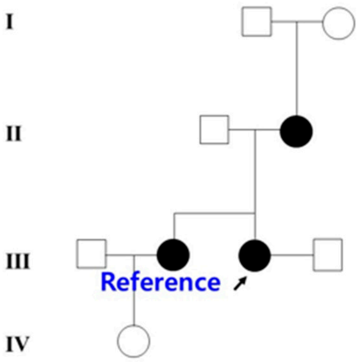


**4. DM1 (DMPK gene)**

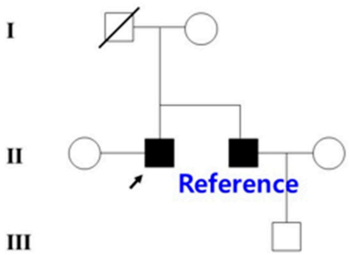


**5. DM1 (DMPK gene)**

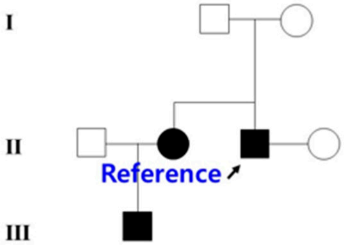


**6. DMD (DMD gene)**

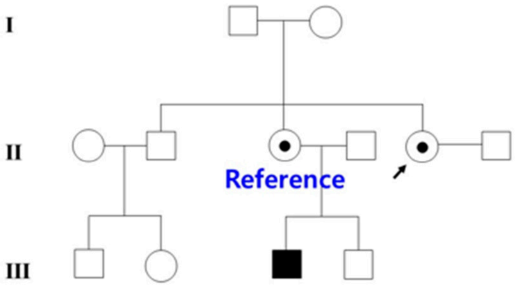

## 7. MFS (FBN1 gene)

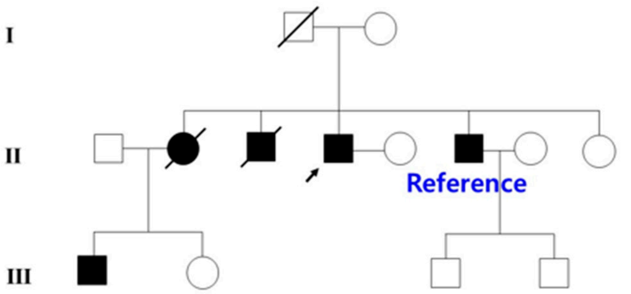


## 8. SCA (ATXN3 gene)

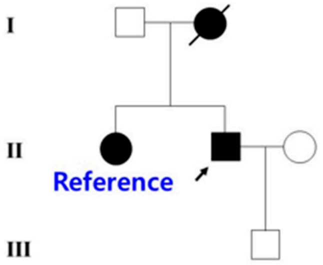


## 9. OTC (OTC gene)

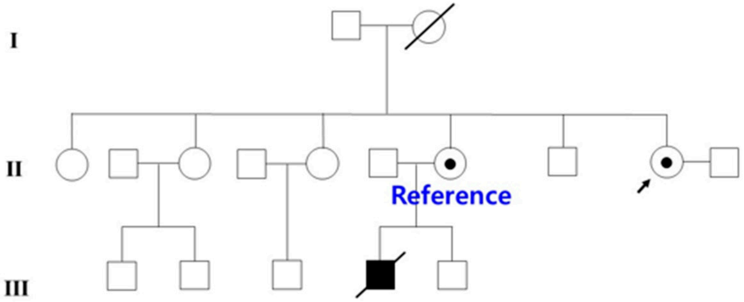


## 10. NF1 (NF1 gene)

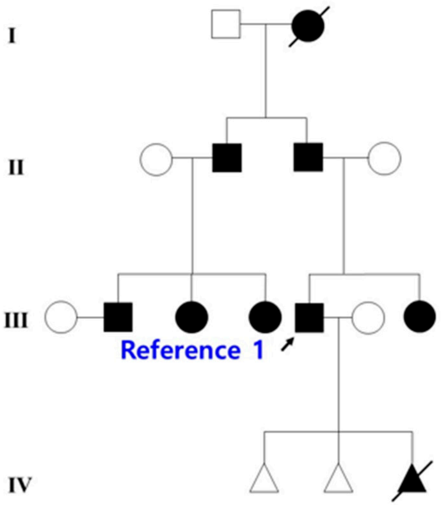


## 11. NF1 (NF1 gene)

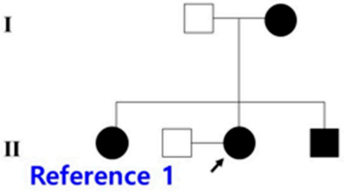


## 12. NF1 (NF1 gene)

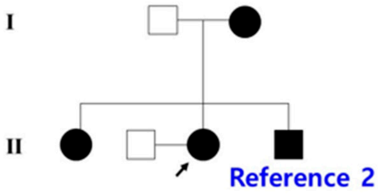

## 13. HEMA (F8 gene)

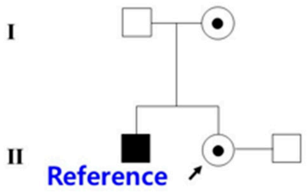


## 14. NF1 (NF1 gene)

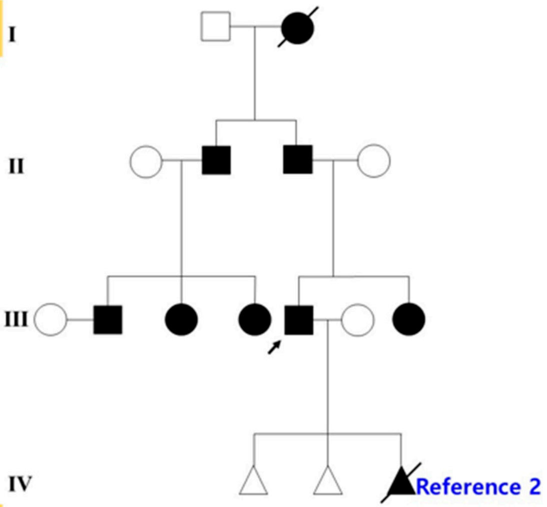


## 15. NF1 (NF1 gene)

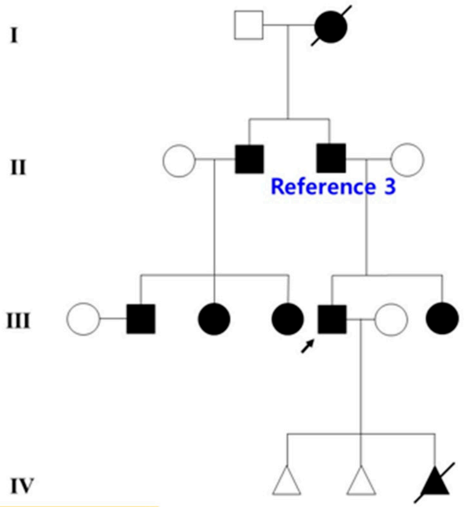


## 16. NF1 (NF1 gene)

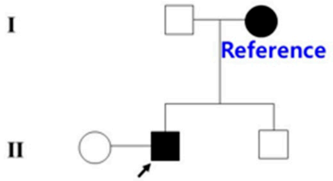


## 17. Fabry disease (GLA gene)

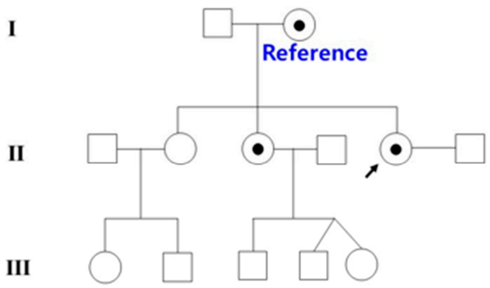


## 18. Kennedy (AR gene)

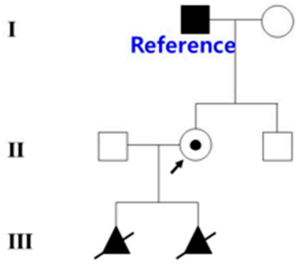

## 19. Kennedy (AR gene)

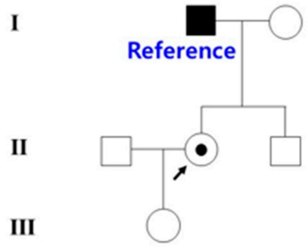


## 20. Kennedy (AR gene)

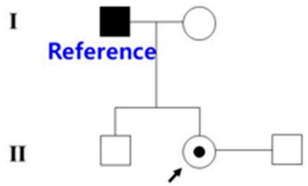


## 21. Kennedy (AR gene)

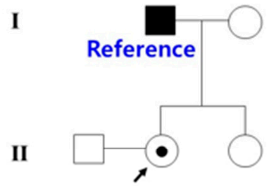


## 22. Kennedy (AR gene)

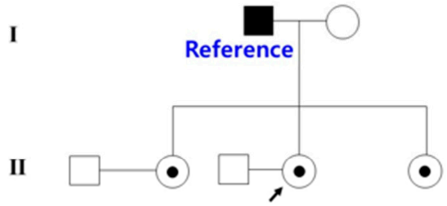

Supplement: Supplementary file 1 [file jpm-15-00273-s001.zip › Figure S1.pdf]
